# Plasma neurofilament light chain and risk of death and stroke in community-dwelling elderly individuals

**DOI:** 10.1093/ehjopen/oeag143

**Published:** 2026-09-16

**Authors:** Julia Aulin, Karl Sjölin, Erika Frank, Johan Lindbäck, Lars Lind, Joachim Burman

**Affiliations:** Department of Medical Sciences, Cardiology, Uppsala University, 751 85 Uppsala, Sweden; Uppsala Clinical Research Center, Uppsala University, Dag Hammarskjölds väg 38, 751 85 Uppsala, Sweden; Department of Medical Sciences, Neurology, Uppsala University, 751 85 Uppsala, Sweden; Uppsala Clinical Research Center, Uppsala University, Dag Hammarskjölds väg 38, 751 85 Uppsala, Sweden; Uppsala Clinical Research Center, Uppsala University, Dag Hammarskjölds väg 38, 751 85 Uppsala, Sweden; Department of Medical Sciences, Cardiology, Uppsala University, 751 85 Uppsala, Sweden; Department of Medical Sciences, Neurology, Uppsala University, 751 85 Uppsala, Sweden

**Keywords:** Neurofilament light chain, Glial fibrillary acidic protein, Elderly

## Abstract

**Aims:**

Circulating neuroinjury biomarkers such as neurofilament light chain (NFL) and glial fibrillary acidic protein (GFAP) have emerged as indicators of brain health. While NFL predicts stroke in patients with atrial fibrillation, its prognostic value in unselected elderly populations is uncertain. We investigated the associations of NFL and GFAP with cardiovascular events and death in community-dwelling older adults.

**Methods and results:**

Baseline plasma NFL and GFAP concentrations were determined using high-sensitivity Simoa® assays in 806 participants aged 75 years from the Prospective Investigation of the Vasculature in Uppsala Seniors (PIVUS) study. Cox regression models with sequential adjustment for demographics, comorbidities, renal function, and NT-proBNP assessed associations with ischaemic stroke, myocardial infarction, cardiovascular death, all-cause death, and major adverse cardiovascular events (MACE; stroke, myocardial infarction, and all-cause death). During a median follow-up of 10 years, 55 ischaemic strokes, 48 myocardial infarctions, 52 cardiovascular deaths, and 188 all-cause deaths occurred. Neurofilament light chain was not independently associated with ischaemic stroke or myocardial infarction and showed no independent association with cardiovascular death after adjustment including NT-proBNP. In contrast, higher NFL was independently associated with all-cause death (adjusted hazard ratio per doubling 1.91; 95% CI 1.49–2.46) and MACE (1.53; 95% CI 1.21–1.93), and improved discrimination for all-cause death beyond established risk factors. Glial fibrillary acidic protein was not associated with any outcome.

**Conclusion:**

In community-dwelling elderly individuals, higher NFL was associated with increased risk of death but not with specific cardiovascular events. These findings suggest that NFL reflects overall physiological vulnerability rather than discrete vascular risk.

## Introduction

Cerebrovascular disease and neurodegeneration are major contributors to morbidity and mortality in older adults. In addition to clinically overt stroke, aging is associated with a substantial burden of subclinical brain injury, including silent cerebral infarcts, white matter disease, and neuroaxonal degeneration, which contribute to cognitive decline, functional impairment, and increased mortality.^1–4^ Assessment of such brain injury has traditionally relied on neuroimaging or cerebrospinal fluid analysis, but recent advances now allow sensitive detection of neuroglial proteins in peripheral blood.^5^

Neurofilament light chain (NFL) is a structural protein of large myelinated axons that is released into the circulation following neuroaxonal injury. Advances in ultrasensitive assay technologies have established circulating NFL as a robust blood-based biomarker of neuronal injury across a broad range of neurological conditions, including ischaemic stroke, cerebral small vessel disease, and neurodegenerative disorders.^6,7^ Circulating NFL concentrations increase with age and have been associated with structural brain changes, cognitive impairment, and adverse clinical outcomes in both neurological and non-neurological populations. Together, these observations suggest that circulating NFL may reflect cumulative neuroaxonal injury driven by multisystem aging and chronic disease, rather than discrete vascular events.

Cardiovascular disease is increasingly recognized as an important contributor to brain injury beyond overt cerebrovascular events. Conditions such as hypertension, heart failure, renal dysfunction, and atrial fibrillation (AF) have been linked to covert cerebral injury and impaired brain health.^8^ In patients with AF, elevated NFL levels have been associated with an increased risk of stroke and death, independent of established clinical risk factors and cardiac biomarkers, supporting the concept that NFL reflects downstream cerebral consequences of systemic disease.^9,10^

However, the clinical significance of circulating NFL in community-dwelling elderly individuals without selected high-risk cardiovascular conditions remains unclear. In particular, it is unknown whether NFL primarily reflects susceptibility to specific vascular events, such as ischaemic stroke, or whether it represents a broader marker of biological aging and vulnerability associated with adverse outcomes. In this study, we investigated plasma NFL levels, and included the astroglial biomarker glial fibrillary acidic protein (GFAP) as an exploratory biomarker reflecting astrocytic activation and injury, in 806 community-dwelling 75-year-old participants in the Prospective Investigation of the Vasculature in Uppsala Seniors (PIVUS) study,^11^ and examined their associations with ischaemic stroke, myocardial infarction, cardiovascular death, all-cause death, and a composite outcome of major adverse cardiovascular events (MACE) including stroke, myocardial infarction, and all-cause death, during long-term follow-up, while accounting for clinical characteristics, comorbidities, and established prognostic biomarkers.

## Methods

### Study design and participants

Plasma samples from 806 community-dwelling 75-year-olds included in the PIVUS study were collected at baseline, and the participants were followed for a median of 10 years. The details of the PIVUS study have previously been published.^11^ Briefly, the PIVUS study was conducted between April 2001 and June 2004, and eligible participants included all men and women aged 70 years residing in the Uppsala community. The subjects were randomly chosen from the register of community living, and subjects received an invitation letter within 2 months of their 70th birthday. Of the 2025 subjects invited, 1016 subjects accepted participation and underwent investigation. Re-investigations were performed at ages 75 and 80. Biomarkers of subclinical cardiovascular disease, including three tests of endothelial function, three tests of arterial compliance, carotid atherosclerosis, left ventricular mass, left ventricular systolic and diastolic function, and heart rate variability, were recorded at the examinations, in addition to genomic, proteomic, and metabolomics data acquired at ages 70, 75, and 80. Cardiovascular outcomes in the original PIVUS cohort were followed for up to 15 years from the age-70 examination. For the present study, baseline was defined as the examination at age 75 years, when samples for NFL and GFAP measurements were obtained, resulting in up to approximately 10 years of follow-up. The study was approved by the Ethics Committee of Uppsala University and the participants gave informed consent.

### Biochemical analyses

The concentrations of NFL were determined in all available plasma samples from the 75-year-old examination (*n* = 806) of patients included in PIVUS.

The concentrations of NFL and GFAP were determined in plasma with the Single Molecule Array (Simoa®) N2PB kit run on a SR-X™ Biomarker Detection System (Quanterix). All analyses were made in batch by the same board-certified technician working at the Department of Clinical Chemistry, Uppsala University Hospital, Uppsala, Sweden. The lower limit of detection (LLOD) for NFL was 0.099 ng/L and the lower limit of quantification was 0.625 ng/L with a dynamic range in plasma of 0–2000 pg/mL. The LLOD for GFAP was 1.0 ng/L and the lower limit of quantification was 9.4 ng/L with a dynamic range in plasma of 0–40 000 pg/mL.

Intra-plate precision was assessed using duplicate measurements of internal controls on each plate and summarized as mean coefficient of variation (CV) ±SD across plates. For GFAP, the mean intra-plate CV was 4.5 ± 4.4% at low concentrations and 4.9 ± 5.0% at high concentrations. For NFL, the corresponding values were 8.8 ± 7.3% at low concentrations and 3.4 ± 3.6% at high concentrations. Inter-plate precision was evaluated using plate-level mean concentrations across all runs. For GFAP, the inter-plate CV was 9.9% at both low and high concentrations. For NFL, inter-plate CVs were 12% at low concentrations and 9.2% at high concentrations.

### Statistical analyses

Baseline characteristics are summarized by sample quartile groups of NFL and for the full cohort using frequencies for categorical variables, and median and quartiles for continuous variables. The distribution of NFL is presented graphically by an empirical cumulative distribution function plot. Neurofilament light chain was log-transformed in all analyses. Times to the following outcomes were studied: ischaemic stroke, myocardial infarction, cardiovascular death, and all-cause death. Time to event was defined to start from the visit date at 75 years. Incidence rates were estimated for all outcomes as the number of events divided by the total follow-up time. Corresponding 95% confidence intervals (CI) for incidence rates were estimated using a gamma distribution. The median follow-up time for each outcome was estimated by the Kaplan–Meier method while censoring for the event.

For the association between NFL or GFAP and other variables, linear regression models with NFL or GFAP as the outcome were used. Both univariable models, including each variable one at a time, and multivariable models, including all variables simultaneously, were fitted. Continuous variables were entered as restricted cubic splines with three knots placed at the respective 10th, 50^th^, and 90th sample percentiles. The relative importance of the variables included in the multivariable model was illustrated by plotting their partial R^2^ values. The correlation between NFL and other biomarkers was assessed using the Spearman’s correlation coefficient.

The cumulative event rate for each outcome, estimated by the Kaplan–Meier method, was plotted for an arbitrary division of NFL into quartiles. Cox-regression models were used to estimate unadjusted and adjusted associations between NFL or GFAP and each outcome. Adjustments were made incrementally in four steps: Model 0: Unadjusted; Model 1: Adjusted for sex + creatinine + body mass index (BMI); Model 2: Model 1 + diabetes + heart failure; Model 3: Model 2 + NT-proBNP. To allow for non-linear associations, NFL and GFAP were first represented as a restricted cubic spline with three knots placed at the 10th, 50th, and 90th sample percentiles, respectively. Since the associations between NFL/GFAP and the log hazard of the outcomes were close to linear, we proceeded to continue assuming linearity. Adjusted hazard radtio (HR), corresponding to a doubling of NFL or GFAP, with 95% confidence interval (CI) was presented. Sensitivity analyses restricted to first-time events of myocardial infarction, ischaemic stroke, and MACE were performed by excluding participants with a history of the corresponding event at baseline. Missing data were handled using complete-case analysis; participants with missing data for any covariate included in a given model were excluded from that analysis. No imputation was performed. The predictors’ relative contributions in the models were measured as the partial chi-square statistic minus the predictor degrees of freedom. The discriminative ability was evaluated using Harrell’s c-index.^12^ To measure the added value of adding NFL or GFAP to each respective model, the fraction of new information (FNI) was calculated. FNI was defined as the part of the likelihood ratio (LR) χ^2^ statistic for the model, including NFL or GFAP that would be lost if NFL or GFAP was removed from the model. The C-index with and without NFL or GFAP as well as the FNI provided by NFL or GFAP, are presented for Models 0–3. Testing whether the change in C-index is statistically significant is equivalent to testing whether NFL or GFAP are significant in the model so *P*-values presented for the hazard ratios being 1.0 should also be referred to for the test of improvement of the C-index.^13^ Data management and statistical analyses were performed and coordinated by Uppsala Clinical Research Center (UCR), Uppsala University, Sweden. All analyses were done using R version 4.2.3.^14^

## Results

### Baseline characteristics and demographics

Baseline characteristics of the study population stratified by quartile groups of plasma NFL and for the full cohort are presented in *Table 1*. Overall, 49% of the patients were men, 12% had diabetes mellitus, and 6% had heart failure. A history of previous stroke was present in 6.5% of the study population.

**Table 1:** Baseline characteristics by neurofilament light chain (NFL) quartile group and combined

NFL	*n*	Q1 3.12–16.71 *n* = 202	Q2 16.71–21.26 *n* = 201	Q3 21.26–27.61 *n* = 201	Q4 27.61–273.02 *n* = 202	Combined *n* = 806
Sex, male	806	93 (46.0%)	100 (49.8%)	102 (50.7%)	100 (49.5%)	395 (49.0%)
Height (cm)	806	168.0 (161.0—176.0)	168.0 (161.0—176.0)	169.0 (161.0—176.0)	168.0 (161.0—174.8)	168.0 (161.0—176.0)
Weight (kg)	806	78.0 (70.0—87.0)	76.0 (69.0—87.0)	74.0 (63.0—83.0)	73.0 (65.0—82.0)	75.0 (67.0—85.0)
BMI (kg/m^2^)	806	27.7 (25.0—29.9)	27.0 (24.4—29.8)	25.6 (22.9—28.8)	25.9 (23.6—28.0)	26.4 (23.9—29.4)
Smoker						
Current smoker	800	14 (7.0%)	12 (6.0%)	11 (5.5%)	13 (6.5%)	50 (6.2%)
Previous smoker	753	88 (46.8%)	67 (35.4%)	81 (42.6%)	89 (47.8%)	325 (43.2%)
Diabetes	803	22 (10.9%)	18 (9.0%)	21 (10.5%)	32 (16.0%)	93 (11.6%)
Previous stroke	806	7 (3.5%)	13 (6.5%)	15 (7.5%)	17 (8.4%)	52 (6.5%)
Previous MI	806	14 (6.9%)	17 (8.5%)	12 (6.0%)	28 (13.9%)	71 (8.8%)
Heart failure	804	9 (4.5%)	9 (4.5%)	16 (8.0%)	13 (6.5%)	47 (5.8%)
SBP (mmHg)	805	148.0 (136.0—162.0)	146.0 (135.0—158.0)	148.0 (136.0—160.0)	148.0 (138.0—160.0)	148.0 (136.0—160.0)
DBP (mmHg)	805	76.0 (70.0—83.5)	78.0 (70.0—84.0)	76.0 (70.0—80.0)	74.0 (68.0—80.0)	76.0 (70.0—82.0)
MMT results	775	29.0 (28.0—30.0)	29.0 (28.0—30.0)	29.0 (28.0—30.0)	29.0 (28.0—30.0)	29.0 (28.0—30.0)
Seven-minute test 1 results	780	0.0 (0.0—1.0)	0.0 (0.0—1.0)	0.0 (0.0—1.0)	0.0 (0.0—1.0)	0.0 (0.0—1.0)
TMT-A results	762	53.0 (41.8—65.0)	53.0 (44.0—67.8)	52.5 (42.2—65.8)	55.0 (43.0—72.8)	53.0 (43.0—68.0)
TMT-B results	753	125.0 (94.0—199.0)	132.0 (102.0—175.0)	127.0 (103.8—179.2)	135.0 (101.0—215.5)	129.0 (101.0—191.0)
GFAP (pg/mL)	806	183.8 (146.8—240.6)	222.8 (180.8—284.9)	259.5 (195.2—315.3)	296.3 (234.5—354.6)	237.0 (179.4—307.6)
NT-proBNP (ng/L)	806	114.6 (68.8—199.7)	109.2 (64.2—198.5)	124.1 (73.7—231.2)	158.8 (98.4—379.0)	125.0 (73.5—234.6)
GDF-15 (ng/L)	803	1081.0 (891.0—1407.0)	1169.5 (967.8—1531.8)	1265.5 (1020.8—1693.8)	1494.0 (1181.0—2343.8)	1244.0 (992.0—1700.0)
cTnI (ng/L)	806	4.5 (3.6—6.0)	4.8 (3.5—6.4)	4.8 (3.5—7.2)	5.6 (3.9—8.4)	4.9 (3.6—7.1)
Cystatin C (mg/L)	795	1.1 (1.0—1.2)	1.1 (1.0—1.2)	1.1 (1.0—1.3)	1.2 (1.1—1.4)	1.1 (1.0—1.3)
Creatinine (µmol/L)	798	72.2 (64.3—81.1)	75.8 (67.0—86.8)	79.5 (69.3—89.3)	83.5 (70.4—99.1)	76.7 (67.3—88.9)
CRP (mg/L)	798	2.0 (1.2—3.8)	2.3 (1.1—3.8)	1.9 (1.0—3.8)	2.2 (1.2—4.4)	2.1 (1.1—4.0)
LDL-cholesterol (mmol/L)	805	3.3 (2.8—3.9)	3.4 (2.8—4.1)	3.2 (2.8—3.9)	3.2 (2.6—3.9)	3.3 (2.7—3.9)

m (a—b) represents median (Q1—Q3). *n* (*P*%) represent frequency (percentage). Percentages computed by group.

CRP, C-reactive protein; cTnI, cardiac troponin I; DBP, diastolic blood pressure; GDF-15, growth differentiation factor 15; GFAP, glial fibrillary acidic protein; LDL, low-density lipoprotein; MI, myocardial infarction; MMT, Mini-Mental Test; NT-proBNP, N-terminal pro-B-type natriuretic peptide; SBP, systolic blood pressure; TMT, Trail Making Test.

### Distribution and associations with clinical characteristics and other biomarkers

At age 75, the median concentration of NFL was 21.3 [interquartile range (IQR)16.7–27.6] pg/mL, and of GFAP 237 (IQR 179.4–307.6) pg/mL. The distribution of NFL was similar between sexes, whereas GFAP was higher in women (*Figure 1*). Higher NFL levels were associated with markers of renal dysfunction and lower BMI, followed by sex (see Supplementary material online, *Figure S1*), whereas higher GFAP levels were associated mainly with sex and renal dysfunction, followed by lower BMI (see Supplementary material online, *Figure S2*). In multivariable analysis, NFL was independently associated with higher creatinine, higher cystatin C, higher NT-proBNP, and lower BMI (see Supplementary material online, *Table S1*). As shown in Supplementary material online, *Figure S3*, plasma NFL correlated weakly with creatinine (r = 0.27), NT-proBNP (r = 0.20) and Cystatin C (r = 0.31).

**Figure 1:**
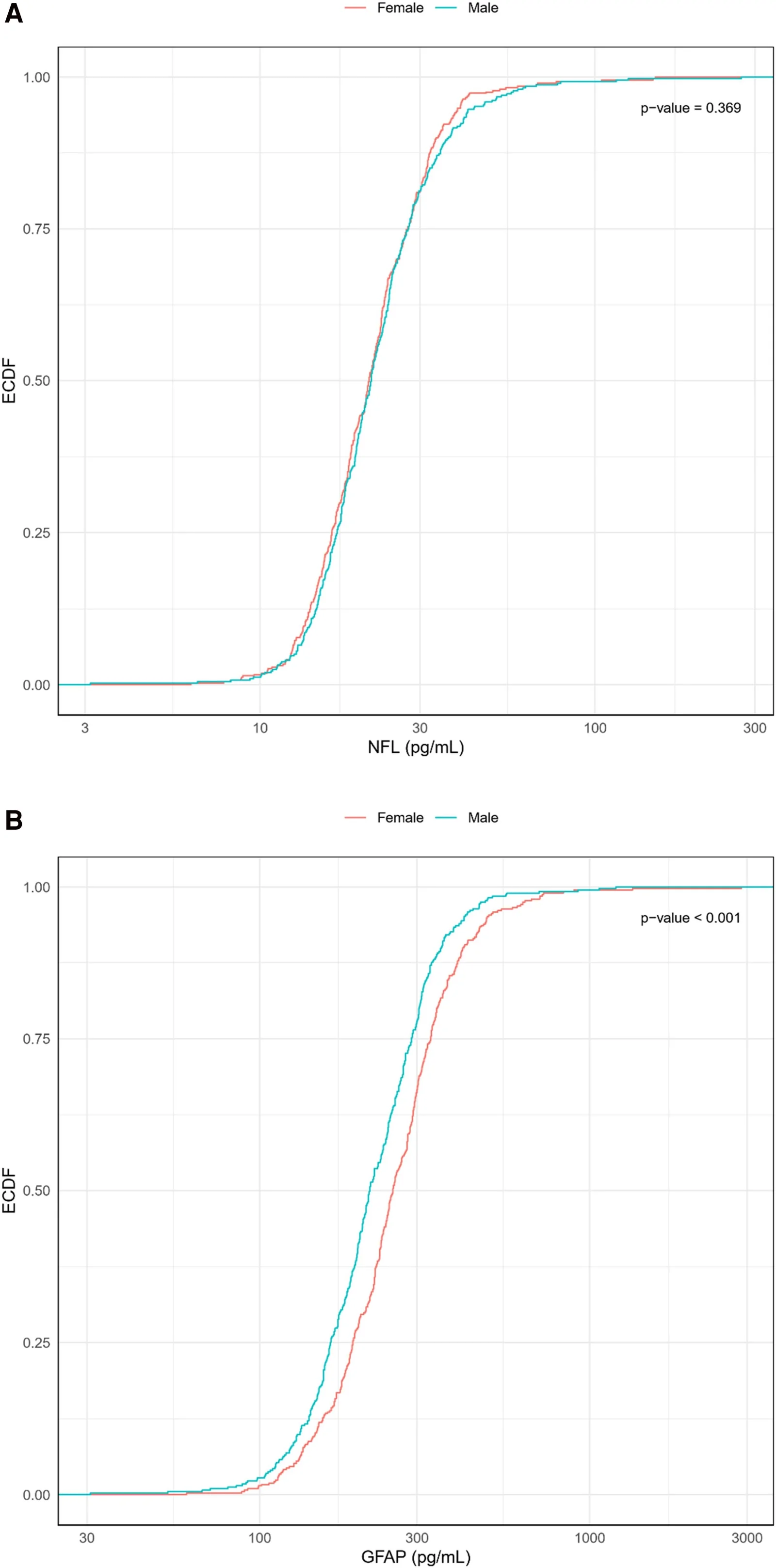
Distribution of NFL and GFAP, respectively, by sex. Distribution of plasma neurofilament light chain (NFL; panel *A*) and glial fibrillary acidic protein (GFAP; panel *B*) concentrations stratified by sex. NFL was log-transformed in all analyses. Biomarker levels are shown as empirical distributions. ECDF = Empirical Cumulative Distribution Function.

### Clinical outcomes during follow-up

Over a median follow-up of 10 (IQR 9.9–10.1) years, a total of 55 ischaemic stroke events, 48 myocardial infarctions, 52 cardiovascular deaths, and 188 deaths from any cause occurred, corresponding to incidence rates of 0.78, 0.67, 0.71, and 2.57 events per 100 person-years, respectively. Associations between plasma NFL and the relative hazard of each outcome are presented in *Figure 2*. Kaplan–Meier estimates of cumulative event rates according to quartiles of NFL are shown in *Figure 3*.

**Figure 2:**
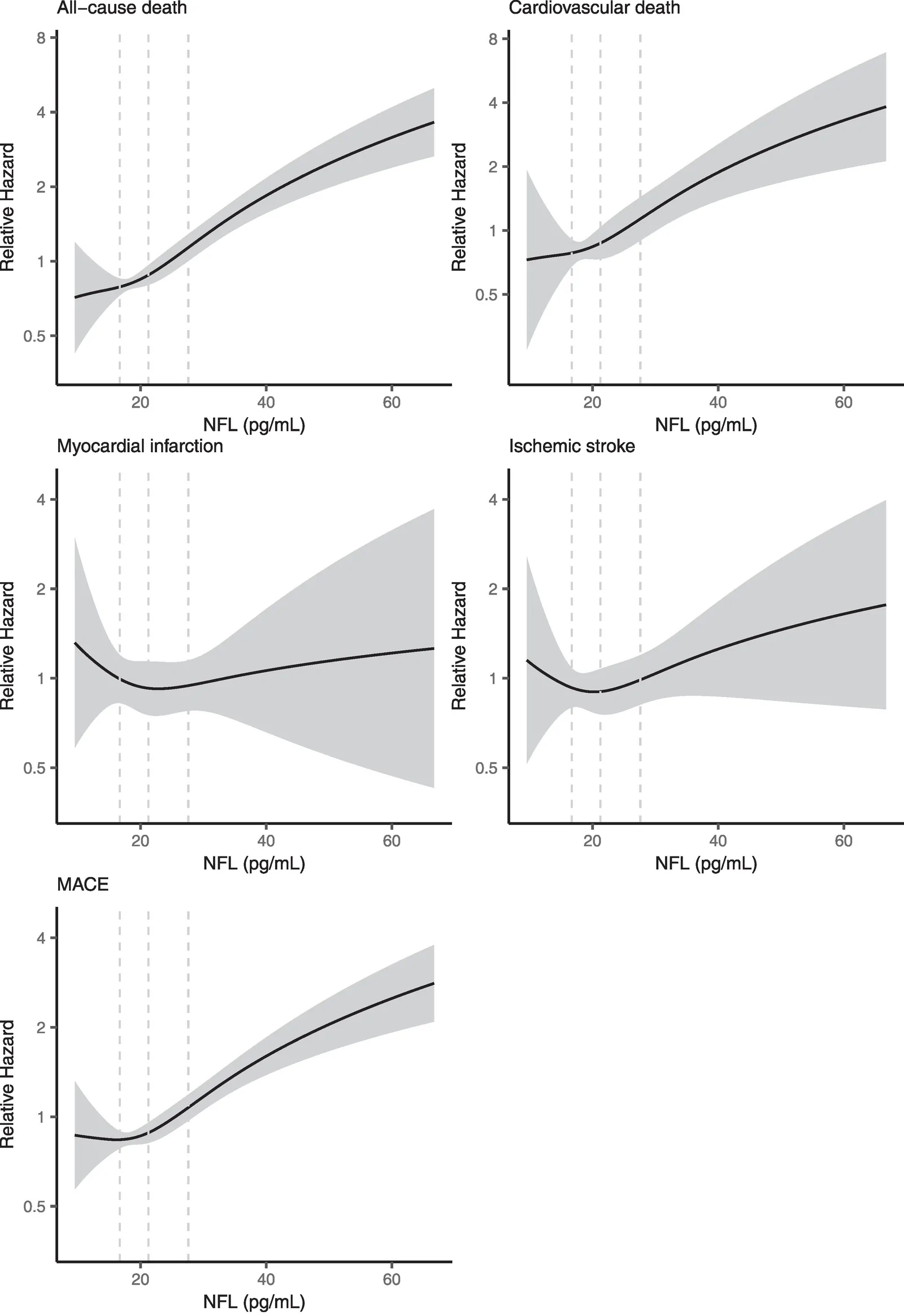
Associations between NFL and outcomes. Associations between plasma neurofilament light chain (NFL) levels and clinical outcomes estimated using Cox proportional hazard models. NFL was modelled using restricted cubic splines with three knots (10th, 50th, and 90th percentiles). Hazard ratios are shown across the distribution of NFL, with dashed vertical lines indicating sample quartiles.

**Figure 3:**
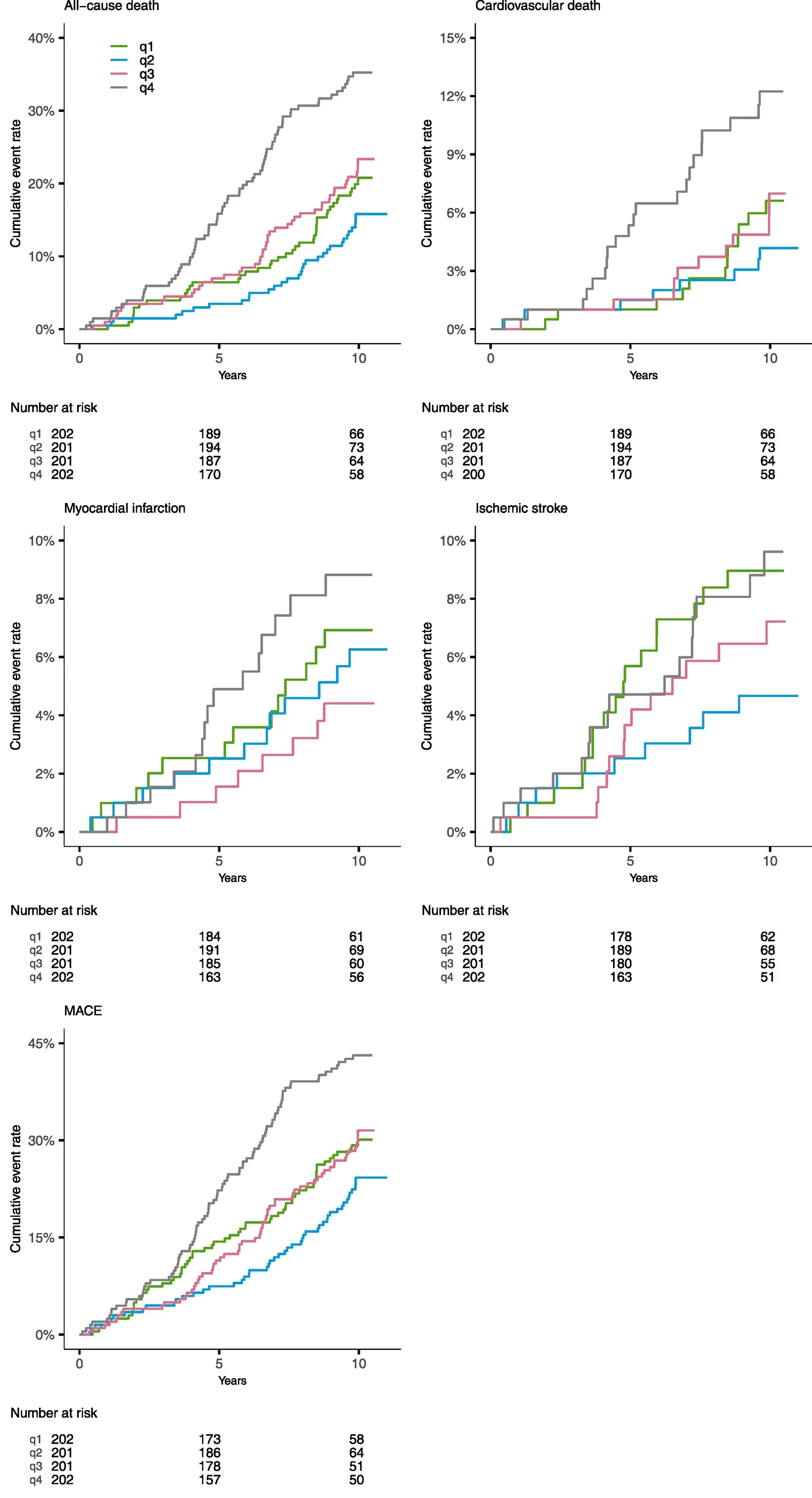
Cumulative incidence of events by NFL quartiles. Kaplan–Meier curves showing cumulative event rates for each outcome stratified by quartiles of plasma neurofilament light chain (NFL) concentration. Time-to-event was calculated from baseline at age 75 years.

### Plasma NFL and GFAP in relation to incident ischaemic stroke

Plasma NFL was not associated with the risk of incident ischaemic stroke. In Cox regression models, there was no evidence of either a linear or non-linear association between NFL and ischaemic stroke in unadjusted analyses or after adjustment for sex, renal function, BMI, diabetes mellitus, heart failure, and NT-proBNP (*Figure 4* and Supplementary material online, *Figure S4*). Kaplan–Meier curves for ischaemic stroke by quartile groups of NFL indicated no consistent separation or positive association over follow-up (*Figure 3*). In sensitivity analyses, excluding participants with a history of stroke at baseline, the association between plasma NFL and first-time ischaemic stroke was similar to that observed in the primary analysis (see Supplementary material online, *Figure S5*).

**Figure 4:**
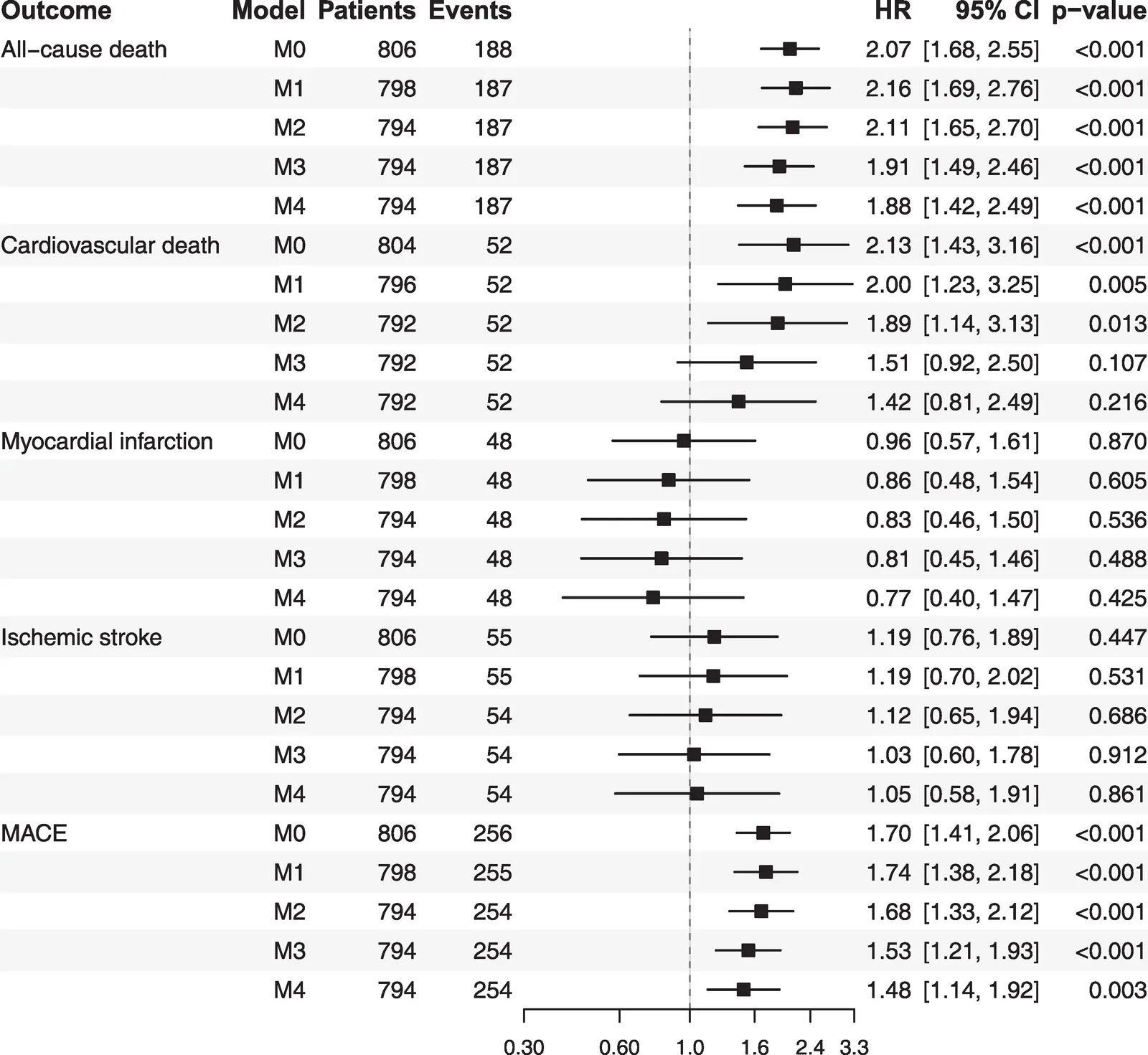
Associations between plasma NFL and clinical outcomes. Forest plot for linear neurofilament light chain (NFL) models showing hazard ratios (HRs) with 95% confidence intervals for the association between plasma NFL and clinical outcomes across sequential Cox regression models (M0–M4). Estimates correspond to a doubling of NFL concentration. Models were adjusted incrementally: M0, unadjusted; M1, adjusted for sex, creatinine, and BMI; M2, additionally adjusted for diabetes and heart failure; M3, additionally adjusted for N-terminal pro-B-type natriuretic peptide (NT-proBNP); M4, fully adjusted model including glial fibrillary acidic protein (GFAP).

Plasma GFAP was also examined in relation to incident ischaemic stroke. Glial fibrillary acidic protein was not associated with ischaemic stroke (*Figure 5*). This lack of association persisted after the further adjustment for vascular risk factors and renal function, as well as after a mutual adjustment for NFL. In the fully adjusted model, GFAP showed no independent association with incident ischaemic stroke (hazard ratio for a doubling of GFAP 0.93, 95% CI 0.57–1.52). No evidence of non-linearity was observed.

**Figure 5:**
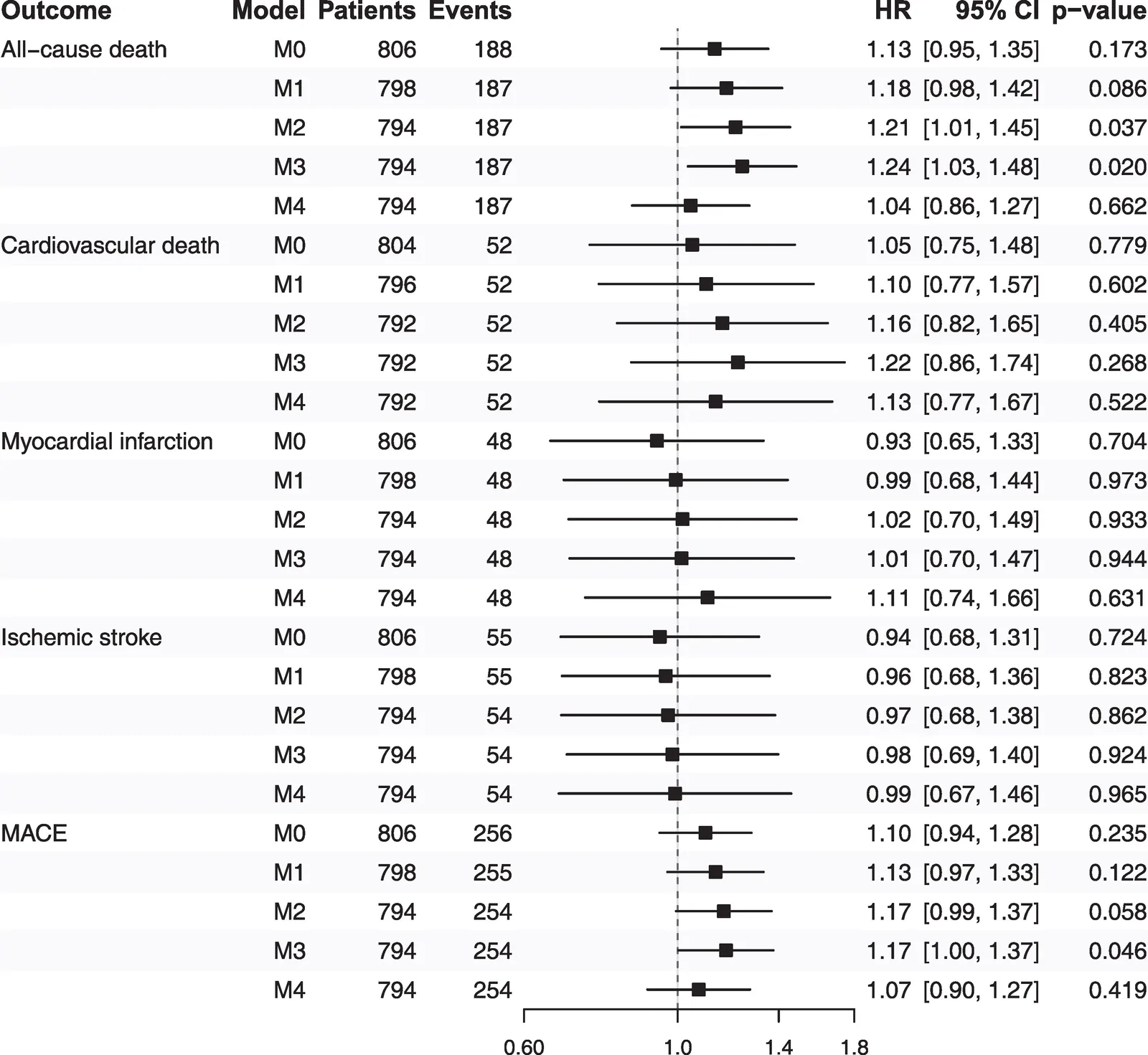
Associations between plasma GFAP and clinical outcomes. Forest plot showing hazard ratios (HRs) with 95% confidence intervals for the association between glial fibrillary acidic protein (GFAP) and clinical outcomes across sequential Cox regression models (M0–M4). Estimates correspond to a doubling of GFAP concentration. Models were adjusted incrementally: M0, unadjusted; M1, adjusted for sex, creatinine, and BMI; M2, additionally adjusted for diabetes and heart failure; M3, additionally adjusted for N-terminal pro-B-type natriuretic peptide (NT-proBNP); M4, fully adjusted model including neurofilament light chain (NFL).

### Plasma NFL and GFAP in relation to incident cardiovascular events

Similarly, NFL was not associated with the risk of myocardial infarction. Higher NFL levels were associated with cardiovascular death in unadjusted models and after adjustment for clinical variables; however, this association was attenuated and no longer statistically significant after additional adjustment for NT-proBNP (hazard ratio for a doubling of NFL 1.51, 95% CI 0.92–2.50) (*Figure 4*). In sensitivity analyses, excluding participants with a history of myocardial infarction at baseline, the association between plasma NFL and first-time myocardial infarction was similar to that observed in the primary analysis (see Supplementary material online, *Figure S5*).

Plasma GFAP was not associated with myocardial infarction or cardiovascular death in any model (*Figure 5*).

### Plasma NFL, GFAP, and all-cause mortality

Higher plasma NFL levels were consistently associated with an increased risk of all-cause death. For a doubling of NFL, the hazard ratio for all-cause death was 2.07 (95% CI 1.68–2.55) in unadjusted analyses and remained significant after adjustment for clinical characteristics, comorbidities, and NT-proBNP 1.91 (95% CI 1.49–2.46) (*Figure 4*). Inclusion of NFL significantly improved discrimination for all-cause mortality. In the fully adjusted model, the C-index increased from 0.693 to 0.707 after addition of NFL.

Plasma GFAP was not associated with all-cause mortality in unadjusted or adjusted Cox regression models (*Figure 5*).

### Plasma NFL, GFAP, and MACE

Higher plasma NFL levels were significantly associated with the composite outcome of MACE consisting of ischaemic stroke, myocardial infarction, and all-cause death, throughout the unadjusted analyses (hazard ratio for a doubling of NFL 1.70, 95% CI 1.41–2.06) to the fully adjusted model including clinical characteristics, comorbidities, and NT-proBNP 1.53 (95% CI 1.21–1.93). In sensitivity analyses, excluding participants with a history of myocardial infarction or stroke at baseline, the association between plasma NFL and first-time MACE was similar to that observed in the primary analysis (see Supplementary material online, *Figure S5*).

Plasma GFAP was not associated with MACE in any of the Cox regression models (*Figure 5*).

## Discussion

In this prospective study of community-dwelling elderly individuals, higher plasma NFL levels were independently associated with all-cause death and MACE but not with incident ischaemic stroke, myocardial infarction, or cardiovascular death after comprehensive adjustment for clinical risk factors and NT-proBNP. In contrast, the astrocytic injury marker GFAP was not associated with any of the investigated outcomes. These findings suggest that circulating NFL reflects processes linked to overall vulnerability and long-term survival rather than serving as a specific risk marker for ischaemic stroke in a relatively healthy elderly population with a low burden of overt cerebrovascular disease.

The absence of an association between plasma NFL and incident ischaemic stroke in the present study contrasts with our previous observations in patients with AF, where elevated NFL predicted stroke and systemic embolism independently of clinical risk factors and established cardiovascular biomarkers.^9,10^ In patients with AF, NFL appears to capture the burden of recurrent overt and covert cerebral ischaemic injury, likely driven by embolic mechanisms and chronic cerebral hypoperfusion.^1,15,16^

In the population-based Rotterdam Study, Heshmatollah *et al.* measured plasma biomarkers in 4661 stroke-free participants and observed 379 first-ever strokes over a mean follow-up of 10.8 years, reporting a significant association between higher NFL and incident stroke (HR 1.27 per 1-SD increase) and an even stronger association for haemorrhagic stroke.^17^ These results support the concept that circulating NFL can reflect cerebrovascular vulnerability in community cohorts. However, important differences may explain why we did not observe a similar association in PIVUS. Our cohort was smaller (*n* = 806) with fewer ischaemic stroke events (*n* = 55), limiting the power to detect modest hazard ratios, and we additionally adjusted for NT-proBNP, a strong integrative biomarker of vascular and cardiac risk that may attenuate biomarker associations. Importantly, although the Rotterdam Study demonstrates a statistically robust association between NFL and stroke, the reported effect size was modest (HR 1.27 per SD), raising questions about its clinical utility for individual risk prediction, particularly in healthier elderly populations.

In contrast to NFL, the astrocytic biomarker GFAP was not associated with ischaemic stroke, cardiovascular events, or all-cause death. Glial fibrillary acidic protein reflects both acute astrocytic injury and chronic astrogliosis and is elevated in conditions characterized by active neuroinflammation or extensive tissue remodelling, such as multiple sclerosis.^18,19^ The absence of an association for GFAP in the present cohort of community-dwelling elderly individuals without overt neurological disease suggests that ongoing astroglial activation or neuroinflammatory processes are not major contributors to the prognostic signal captured by circulating NFL. The different associations observed for NFL and GFAP may partly reflect differences in the biological processes captured by the two biomarkers. Glial fibrillary acidic protein may reflect both astroglial injury and astrocytic responses to cerebral injury. Furthermore, the biomarkers show different temporal profiles following acute brain injury: after ischaemic stroke, GFAP increases rapidly^20,21^ and peaks within the first few days, whereas NFL increases more gradually and may remain elevated for a prolonged period.^20,22^ Thus, clinically silent or subtle ischaemic brain injury occurring before biomarker sampling may be more likely to be reflected by persistent NFL elevation, whereas a transient GFAP response may already have subsided. This could partly explain the association of NFL, but not GFAP, with subsequent stroke in our study. This also supports the interpretation that NFL primarily reflects cumulative neuroaxonal injury.

The strong relationship between NFL and all-cause death and MACE invites the consideration of its biological meaning beyond the domain of overt neurological disease. In the PIVUS dataset, NFL showed its strongest associations with markers of renal and cardiac dysfunction, including creatinine, cystatin C, and NT-proBNP, and with lower BMI, which may reflect frailty. These associations were evident in both univariable and multivariable models and are consistent with prior studies describing NFL as not only a marker of focal brain injury but also a sensitive indicator of global physiological decline, multisystem disease burden, and biological aging.^6,23–26^ Neuroaxonal injury may occur secondary to systemic vascular disease, chronic inflammation, impaired renal function, or cumulative microvascular damage, all of which are processes linked to aging and mortality. Thus, while NFL did not identify individuals at risk of specific cardiovascular events in this study, it may nonetheless serve as a biomarker that captures broad vulnerability across multiple organ systems and reflects a biological aging trajectory that carries prognostic relevance for death from both cardiovascular and non-cardiovascular causes.

These observations may have important clinical implications. Although no association between NFL and ischaemic stroke was observed in this study, our findings suggest that the relationship between circulating NFL and stroke risk may be weaker in a relatively healthy elderly population than has been reported in cohorts with a higher burden of cardioembolic disease, such as patients with AF. This supports the concept that the prognostic value of NFL is context-dependent and shaped by underlying disease mechanisms. In contrast to ischaemic stroke, the strong and independent association between NFL and all-cause death as well as MACE indicates that NFL may be more informative as a marker of global vulnerability and biological aging rather than as a predictor of specific cardiovascular events. From a clinical perspective, NFL could therefore contribute to risk assessment strategies aimed at identifying vulnerable older adults or individuals with reduced physiological reserve. Whether serial measurements of NFL, rather than a single baseline value, could enhance its prognostic utility or help distinguish reversible from progressive neuroaxonal injury warrants further investigation.

The strengths of this study include the prospective design, the well-characterized cohort of community-dwelling older adults, and the long duration of follow-up. The use of a relatively healthy elderly population provides insight into the prognostic value of NFL outside high-risk clinical settings, thereby complementing prior studies in selected patient populations. A limitation is that the relationship between NFL and subclinical brain pathology in healthy elderly individuals remains largely unexplored, as brain imaging data were not included in this analysis. Consequently, we cannot determine whether elevated NFL levels reflected silent cerebrovascular disease, neurodegeneration, or other structural brain changes.

## Conclusions

In community-dwelling elderly individuals, higher plasma NFL levels were independently associated with all-cause death and MACE, but not with ischaemic stroke, myocardial infarction, or cardiovascular death after accounting for clinical characteristics, comorbidities, and NT-proBNP. These findings suggest that NFL primarily serves as a marker for global physiological vulnerability, rather than a specific predictor of cerebrovascular events in this population. Further research is warranted to clarify the biological pathways linking NFL to aging and mortality and to determine whether integration of NFL into multimarker strategies could improve risk stratification in geriatric care.

## Lead author biography


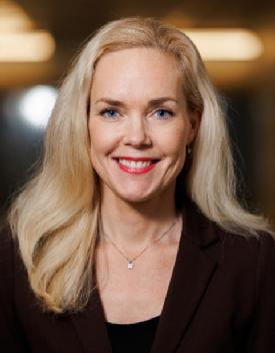
 **Julia Aulin, MD, PhD**, is a consultant cardiologist at the Department of Cardiology, Uppsala University Hospital and a postdoctoral researcher at Uppsala University and Uppsala Clinical Research Center (UCR), Sweden. Her research focuses on cardiovascular diseases, atrial fibrillation, stroke prevention, and the heart–brain axis, with an emphasis on risk prediction and precision medicine using circulating biomarkers. She is involved in national and international research collaborations and uses epidemiological cohorts and large-scale clinical trial datasets to investigate the links between cardiovascular disease and brain health.

## Supplementary Material

oeag143_Supplementary_Data

## Data Availability

The data underlying this article are not publicly available to preserve individuals’ privacy under the European General Data Protection Regulation. A fully anonymized dataset supporting this article is available upon reasonable request to the corresponding author.
