# Supplementary material for: Plasma neurofilament light chain and risk of death and stroke in community-dwelling elderly individuals
Source: Eur Heart J Open. 2026 Sep 16;6(5):oeag143. doi: 10.1093/ehjopen/oeag143 (PMC13689698; doi:10.1093/ehjopen/oeag143)
Supplement: Supplementary file 1 [file oeag143_supplementary_data.pdf]

SUPPLEMENTARY MATERIAL

Plasma Neurofilament Light Chain and Risk of Death and Stroke in Community-dwelling Elderly Individuals

Aulin J, Sjölin K, Frank E, Lindbäck J, Lind L, Burman J

Contents

Table S1. 2
Figure S1. 3
Figure S2. 4
Figure S3. 5
Figure S4 6
Figure S5. 7

Table S1. Associations between NFL and baseline variables.

			Univariable		Multivariable					
Variable	Low	High	Ratio [95% CI]	p-value	Ratio [95% CI]	p-value	Ratio [95% CI]	p-value	Ratio [95% CI]	p-value
Sex	Female	Male	1.03 [0.974, 1.091]	0.299	0.89 [0.835, 0.943]	<0.001	0.92 [0.863, 0.983]	0.013	0.97 [0.913, 1.030]	0.313
BMI (kg/m^2)	23.9	29.4	0.93 [0.900, 0.971]	0.002	0.90 [0.871, 0.939]	<0.001	0.90 [0.868, 0.939]	<0.001	0.93 [0.892, 0.959]	<0.001
Current smoker	No	Yes	1.01 [0.894, 1.131]	0.927	0.89 [0.541, 1.470]	0.653	0.89 [0.545, 1.439]	0.623	1.01 [0.646, 1.572]	0.974
Previous smoker	No	Yes	1.03 [0.970, 1.091]	0.344	1.02 [0.971, 1.080]	0.379	1.03 [0.976, 1.084]	0.292	1.02 [0.974, 1.072]	0.374
Diabetes	No	Yes	1.11 [1.015, 1.213]	0.022	1.09 [1.004, 1.185]	0.040	1.02 [0.938, 1.115]	0.608	1.07 [0.987, 1.158]	0.101
Heart failure	No	Yes	1.20 [1.062, 1.353]	0.003	1.10 [0.986, 1.237]	0.085	1.01 [0.897, 1.131]	0.903	0.99 [0.891, 1.103]	0.877
SBP (mmHg)	136	160	1.01 [0.973, 1.045]	0.654	1.05 [1.009, 1.090]	0.048	1.03 [0.996, 1.076]	0.123	1.02 [0.985, 1.057]	0.348
DBP (mmHg)	70	82	0.96 [0.924, 0.994]	0.071	0.94 [0.905, 0.979]	0.008	0.95 [0.915, 0.988]	0.029	0.96 [0.930, 0.998]	0.078
Creatinine (µmol/L)	4.2	4.5	1.15 [1.105, 1.189]	<0.001	1.21 [1.159, 1.260]	<0.001	1.09 [1.032, 1.153]	0.001	1.10 [1.043, 1.155]	<0.001
NT-proBNP (ng/L)	3.1	7.7	1.10 [1.063, 1.144]	<0.001			1.03 [0.995, 1.072]	0.146	1.04 [1.007, 1.078]	0.021
GDF-15 (ng/L)	6.5	8.4	1.17 [1.127, 1.223]	<0.001			1.10 [1.051, 1.148]	<0.001	1.07 [1.032, 1.119]	<0.001
cTnI (ng/L)	0.6	4.1	1.06 [1.023, 1.104]	0.001			1.00 [0.959, 1.035]	0.588	1.00 [0.965, 1.035]	0.992
Cystatin C (mg/L)	-0.2	0.8	1.16 [1.119, 1.196]	<0.001			1.08 [1.027, 1.133]	0.003	1.05 [1.003, 1.098]	0.048
CRP (mg/L)	-1.3	3.2	1.01 [0.979, 1.051]	0.310			0.99 [0.952, 1.019]	0.679	0.99 [0.956, 1.018]	0.688
LDL-cholesterol (mmol/L)	0.3	1.7	0.98 [0.941, 1.014]	0.408			1.02 [0.987, 1.060]	0.422	1.03 [0.999, 1.066]	0.143
GFAP (pg/mL)	4.5	6.6	1.25 [1.213, 1.292]	<0.001					1.20 [1.166, 1.239]	<0.001

SBP, systolic blood pressure; DBP, diastolic blood pressure; NT-proBNP, N-terminal pro-B-type natriuretic peptide; GDF-15, growth differentiation factor 15; cTnI, cardiac troponin I; CRP, C-reactive protein; LDL, low-density lipoprotein; GFAP, glial fibrillary acidic protein.

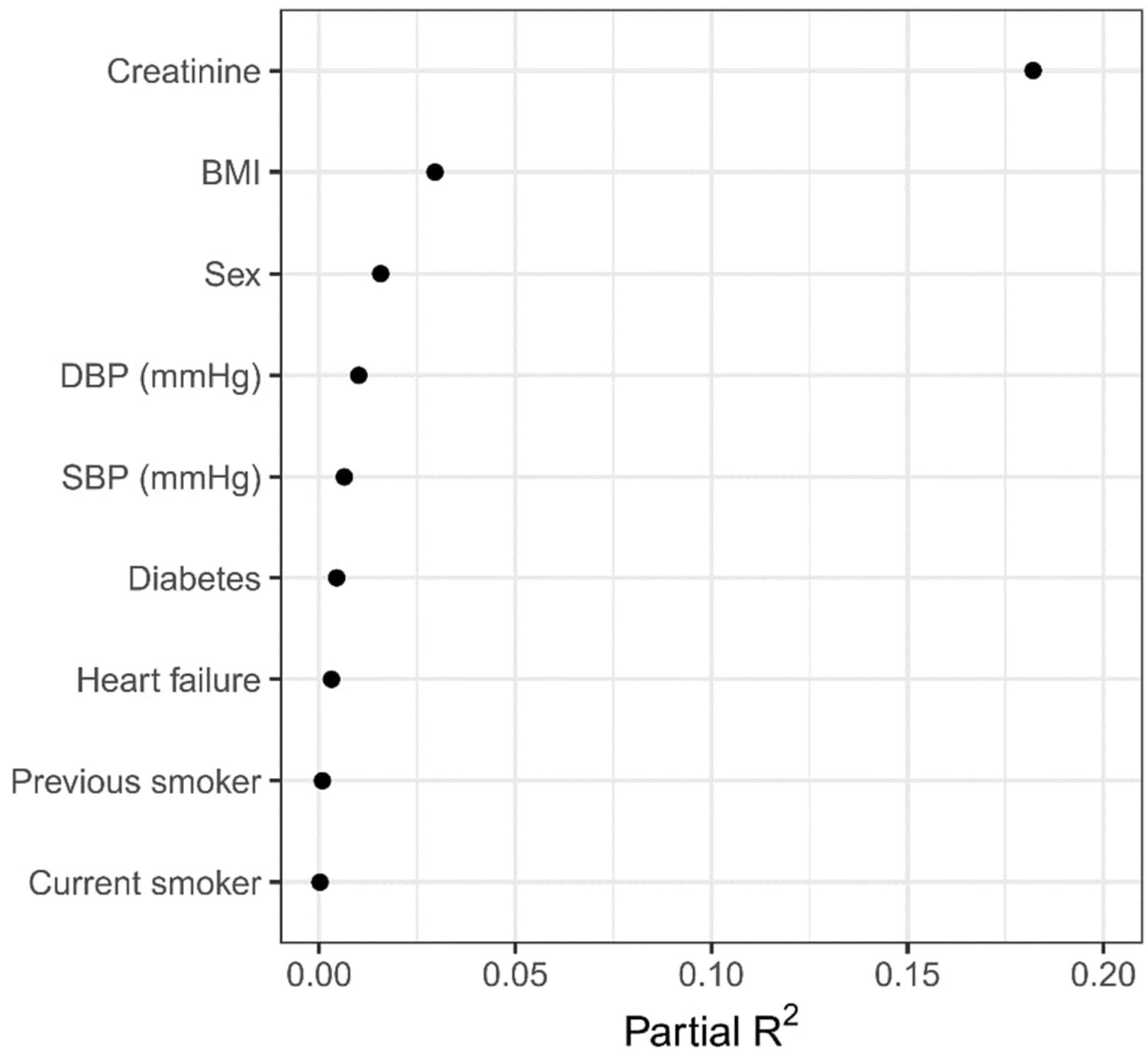

Figure S1. Determinants of plasma NFL levels.

Relative contribution of clinical variables to plasma neurofilament light chain (NFL) levels in a multivariable linear regression model. Variable importance is expressed as partial R^2, with total model R^2 = 0.238. DBP, diastolic blood pressure; SBP, systolic blood pressure.

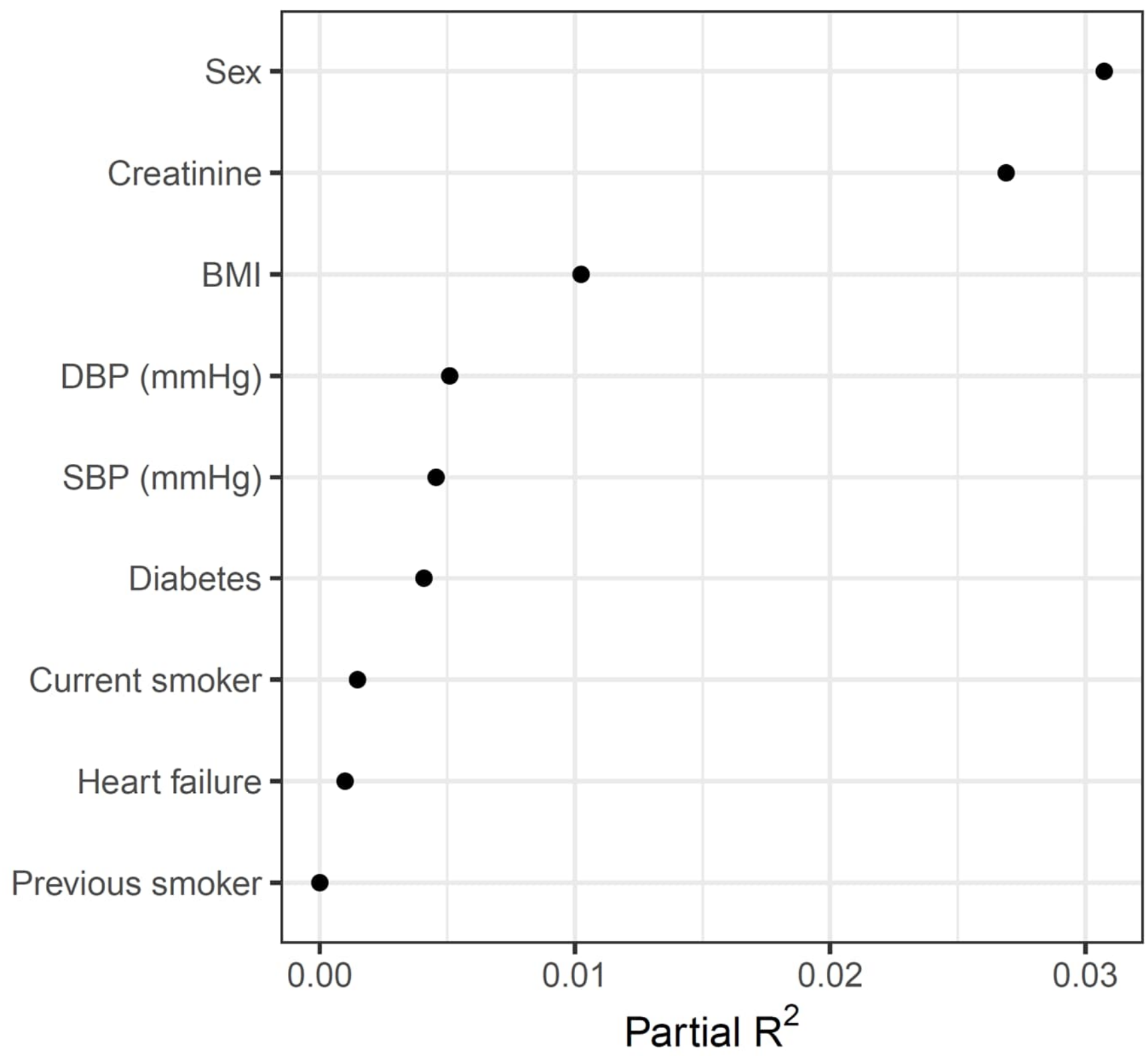

Figure S2. Determinants of plasma GFAP levels.

Relative contribution of clinical variables to plasma glial fibrillary acidic protein (GFAP) levels in a multivariable linear regression model. Variable importance is expressed as partial R^2, with total model $R^2 = 0.08$. DBP, diastolic blood pressure; SBP, systolic blood pressure.

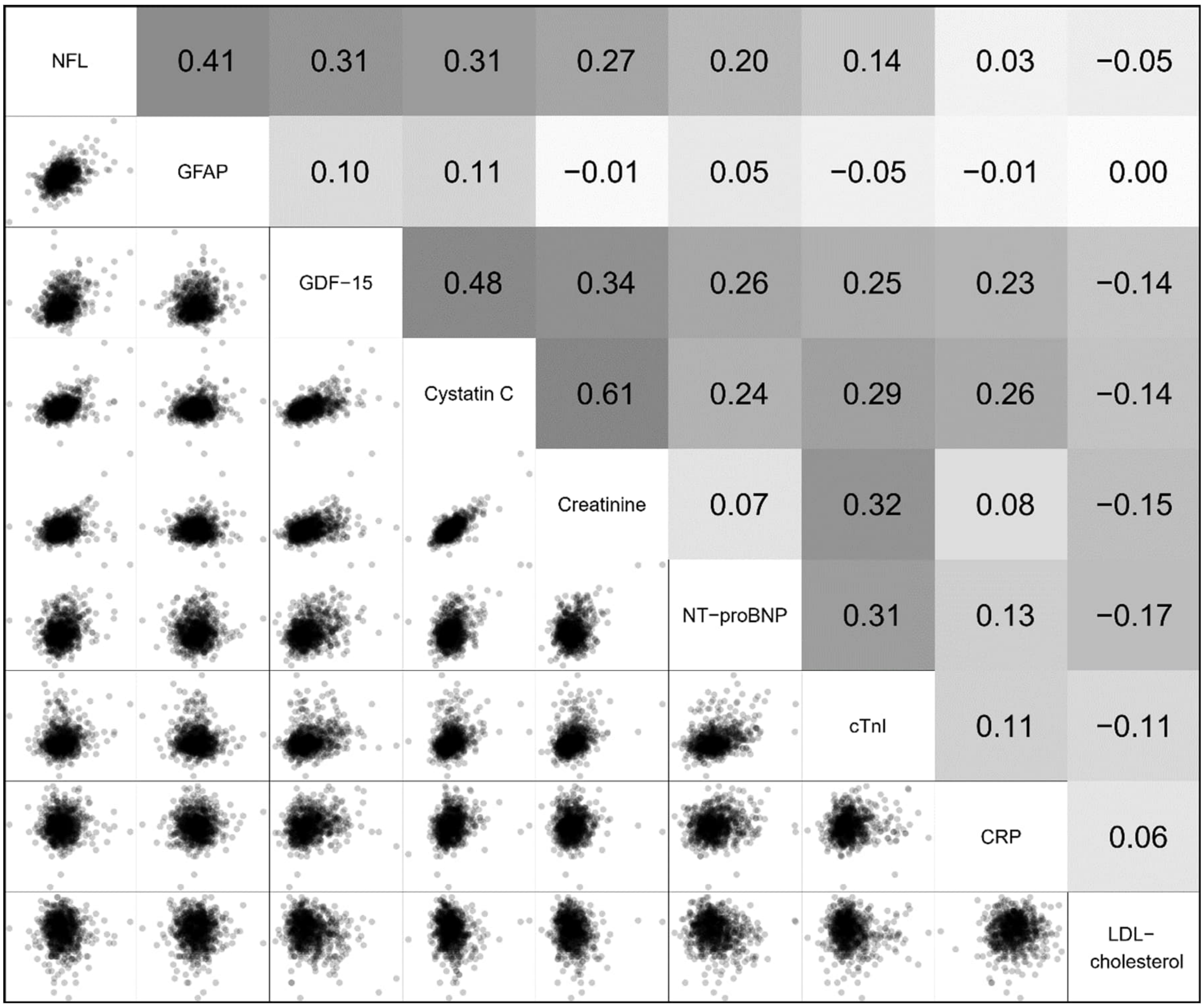

Figure S3. Correlation between NFL and other biomarkers (log-scale).

Scatterplots illustrating the correlation between plasma neurofilament light chain (NFL) concentrations and other circulating biomarkers. Correlation coefficients were estimated using Spearman's rank correlation. GFAP, glial fibrillary acidic protein; GDF-15, growth differentiation factor 15; NT-proBNP, N-terminal pro-B-type natriuretic peptide; cTnI, cardiac troponin I; CRP, C-reactive protein; LDL, low-density lipoprotein.

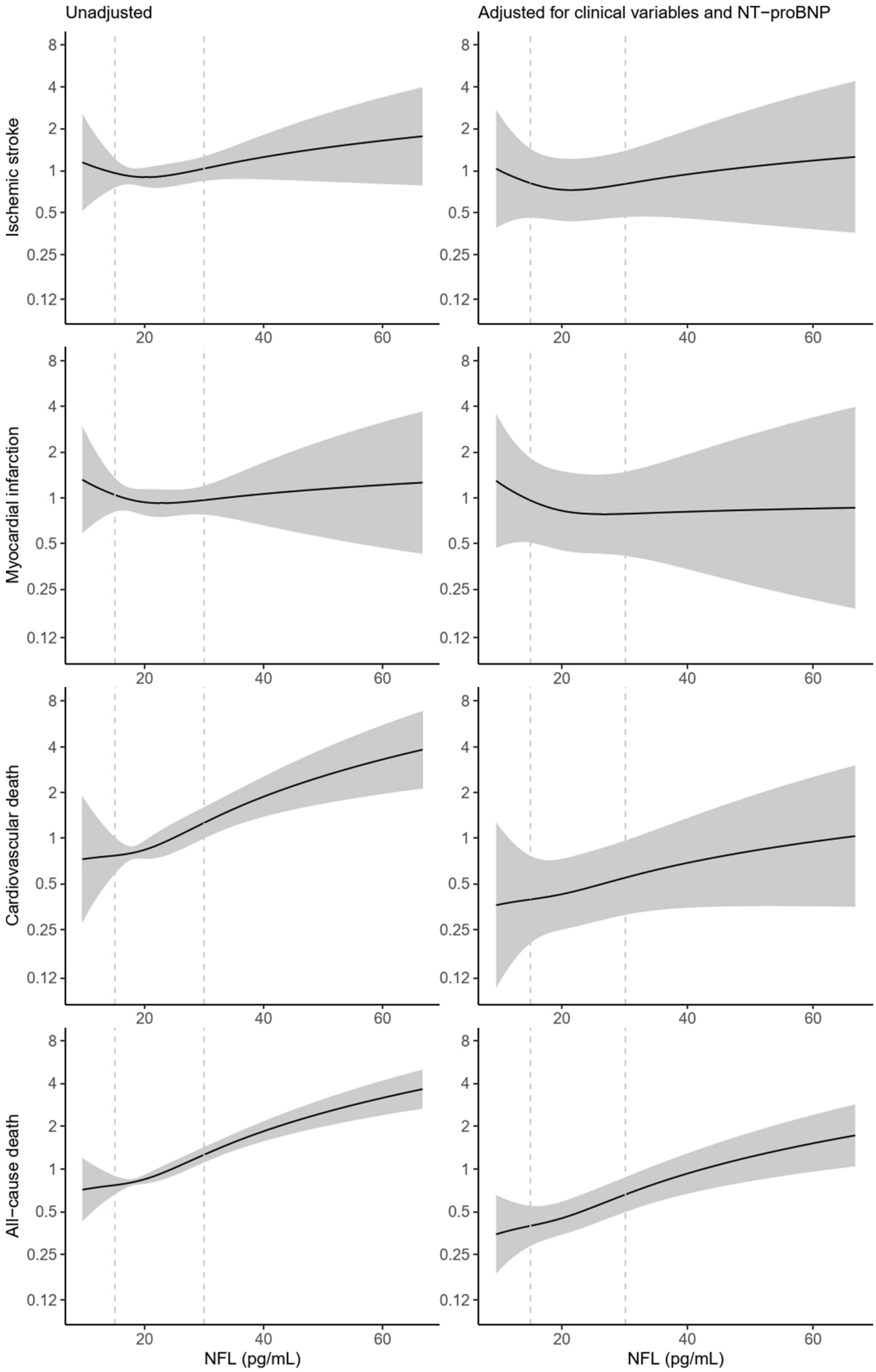

Figure S4. Predicted event rates of the outcomes from Model 3, adjusted for sex, creatinine, BMI, diabetes, heart failure, and N-terminal pro-B-type natriuretic peptide (NT-proBNP). The dashed vertical lines display the values of neurofilament light chain (NFL) used to estimate the reported hazard ratios.

Outcome	Model	Patients	Events	HR	95% CI	p−value
All−cause death	M0	806	188	2.07	[1.68, 2.55]	<0.001
	M1	798	187	2.16	[1.69, 2.76]	<0.001
	M2	794	187	2.11	[1.65, 2.70]	<0.001
	M3	794	187	1.91	[1.49, 2.46]	<0.001
	M4	794	187	1.88	[1.42, 2.49]	<0.001
Cardiovascular death	M0	804	52	2.13	[1.43, 3.16]	<0.001
	M1	796	52	2.00	[1.23, 3.25]	0.005
	M2	792	52	1.89	[1.14, 3.13]	0.013
	M3	792	52	1.51	[0.92, 2.50]	0.107
	M4	792	52	1.42	[0.81, 2.49]	0.216
Myocardial infarction	M0	735	44	0.83	[0.47, 1.45]	0.507
	M1	728	44	0.75	[0.39, 1.42]	0.370
	M2	724	44	0.72	[0.38, 1.39]	0.335
	M3	724	44	0.70	[0.37, 1.34]	0.282
	M4	724	44	0.63	[0.31, 1.27]	0.196
Ischemic stroke	M0	754	54	1.16	[0.72, 1.87]	0.537
	M1	747	54	1.29	[0.74, 2.24]	0.374
	M2	743	53	1.21	[0.68, 2.13]	0.521
	M3	743	53	1.12	[0.64, 1.96]	0.698
	M4	743	53	1.10	[0.59, 2.04]	0.764
MACE	M0	691	203	1.65	[1.31, 2.06]	<0.001
	M1	685	203	1.85	[1.42, 2.42]	<0.001
	M2	681	202	1.81	[1.39, 2.35]	<0.001
	M3	681	202	1.69	[1.30, 2.20]	<0.001
	M4	681	202	1.62	[1.20, 2.19]	0.002

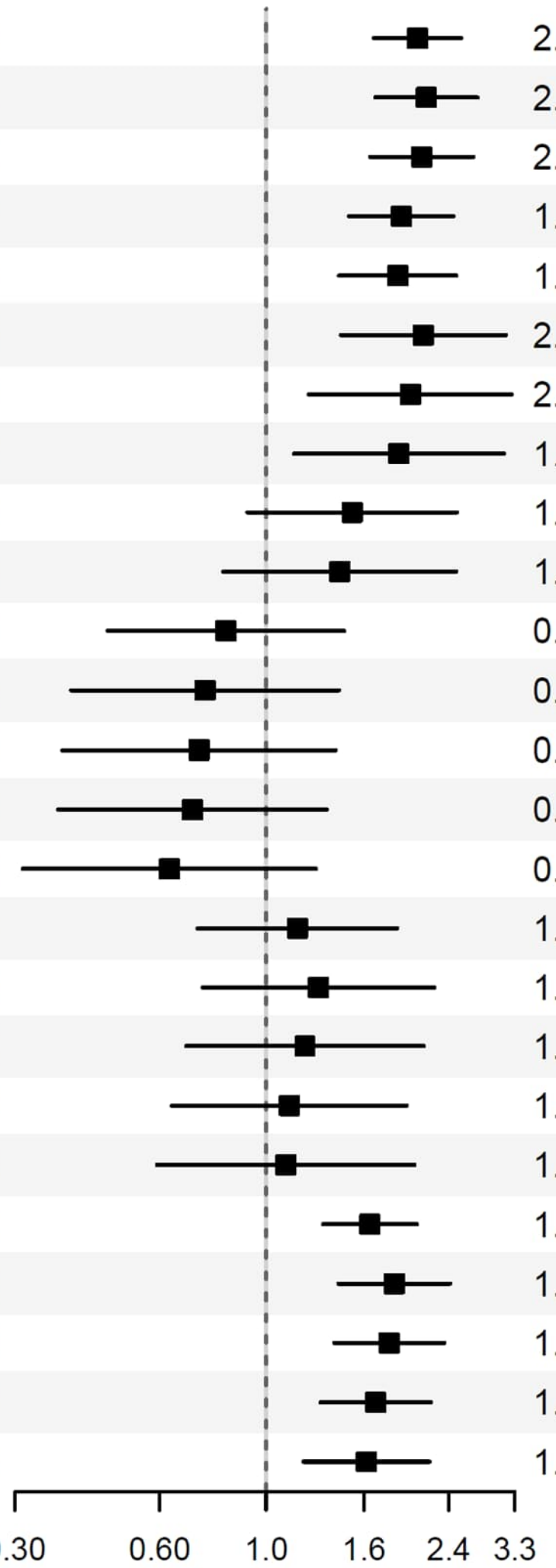

Figure S5. Sensitivity analyses of plasma NFL in relation to first-time myocardial infarction, first-time ischemic stroke, and first-time MACE. Forest plot showing hazard ratios (HRs) with 95% confidence intervals for the association between neurofilament light chain (NFL) and clinical outcomes across sequential Cox regression models (M0–M4) after exclusion of participants with a history of the corresponding outcome at baseline. Hazard ratios are presented per doubling of plasma NFL concentration. Models were adjusted as in the primary analyses: M0, unadjusted; M1, adjusted for sex, creatinine, and BMI; M2, additionally adjusted for diabetes and heart failure; M3, additionally adjusted for N-terminal pro-B-type natriuretic peptide (NT-proBNP); M4, fully adjusted model including neurofilament light chain (NFL).
